# Multidisciplinary Management of Paraneoplastic Anti-N-Methyl-D-Aspartate Receptor Encephalitis Associated With Ovarian Teratoma: A Case Report

**DOI:** 10.7759/cureus.110040

**Published:** 2026-06-01

**Authors:** Ana Paulina Meléndez-Fernandez, María F Ramos-Griera, Pedro Castorena-García, José M Álvarez-Blanco, Roberto De La Peña-López, Nalleli Duran-López, Luis A Tirado-García, Victor Hugo Gomez-Arias, Karla B Molina-Tabarez

**Affiliations:** 1 Surgical Oncology, Médica Sur, Mexico City, MEX; 2 General Surgery, Médica Sur, Mexico City, MEX; 3 Neurology, Médica Sur, Mexico City, MEX; 4 Hematology, Médica Sur, Mexico City, MEX; 5 Oncology, Instituto Nacional de Ciencias Médicas y Nutrición Salvador Zubirán, Mexico City, MEX; 6 Oncology, Médica Sur, Mexico City, MEX; 7 Pathology, Médica Sur, Mexico City, MEX; 8 Neurology, Centro Médico Nacional 20 de Noviembre, Mexico City, MEX

**Keywords:** anti-nmda receptor encephalitis, autoimmune encephalitis, ovarian teratoma, paraneoplastic syndrome, tumor resection

## Abstract

Anti-N-methyl-D-aspartate (NMDA) receptor encephalitis is a severe autoimmune encephalitis often associated with ovarian teratomas and characterized by a rapidly progressive neuropsychiatric syndrome, seizures, dyskinesias, a decreased level of consciousness, and autonomic instability. Antibodies targeting the GluN1 subunit lead to internalization of synaptic NMDA receptors, a mechanism associated with potential neurological recovery when treatment is initiated in a timely manner. Early recognition and initiation of immunotherapy, together with tumor resection, play a key role in improving patient outcomes.

We present the case of a 20-year-old Hispanic woman with subacute neuropsychiatric deterioration and focal seizures beginning in late November 2025, in whom diagnostic evaluation revealed anti-NMDA receptor antibodies and a large ovarian mature cystic teratoma. Due to high clinical suspicion, early surgical resection (<72 hours) and first-line immunotherapy were initiated before antibody confirmation. Following stepwise escalation to plasma exchange, IV immunoglobulin, and second-line therapy with rituximab, the patient demonstrated gradual and sustained neurological recovery after five weeks of inpatient treatment.

This case highlights the importance of a multidisciplinary approach in the management of paraneoplastic anti-NMDA receptor encephalitis and supports the safety of ultra-early surgical resection in tumor-associated cases.

## Introduction

Anti-N-methyl-D-aspartate (NMDA) receptor encephalitis is a severe and potentially reversible autoimmune neurological disorder mediated by IgG antibodies directed against the GluN1 subunit of the NMDA receptor. It is among the most frequently identified forms of autoimmune encephalitis, particularly affecting children and young adults, with a marked female predominance [1,2].

Clinically, the disease follows a characteristic multiphasic course, typically beginning with psychiatric and behavioral symptoms, followed by seizures, hyperkinetic movement disorders, impaired level of consciousness, and autonomic instability [3]. Anti-NMDA receptor antibodies induce cross-linking and internalization of synaptic NMDA receptors without complement-mediated neuronal destruction, explaining the potential for neurological recovery with timely treatment.

A defining feature of this condition is its association with neoplasms in up to 59% of cases, most commonly ovarian teratomas in young women. Neural tissue within teratomas expressing NMDA receptors is believed to act as the antigenic source sustaining the autoimmune response [4-6]. Consequently, tumor resection is not merely diagnostic but also constitutes a disease-modifying intervention.

Early recognition remains challenging because of the heterogeneous clinical presentation and frequently normal neuroimaging findings. CSF analysis often demonstrates only mild inflammatory changes [4]. Current diagnostic frameworks emphasize that immunotherapy should not be delayed while awaiting antibody confirmation in patients with high clinical suspicion and rapid neurological deterioration.

Optimal management requires a multidisciplinary approach involving immunotherapy, neurology, critical care, and surgical or gynecologic oncology. Early tumor removal combined with prompt immunotherapy significantly improves functional outcomes and reduces relapse rates [5]. Timely tumor resection, defined as surgical intervention within the first four weeks after the onset of neurological symptoms, has been associated with reduced exposure to pathogenic autoantibodies, thereby limiting permanent neurological damage and sequelae [7].

This case report aims to highlight the relevance of early tumor identification and resection within the broader multidisciplinary management of anti-NMDA receptor encephalitis, illustrating how timely intervention can critically influence neurological outcomes.

## Case presentation

A previously healthy 20-year-old Hispanic woman developed progressive behavioral changes, anxiety, insomnia, and mood lability over one week in late November 2025, followed by focal seizures, dysarthria, and fluctuating level of consciousness. The clinical course followed the characteristic multiphasic evolution described in anti-NMDA receptor encephalitis, progressing from psychiatric symptoms to seizures, movement disorders, autonomic instability, and impaired consciousness.

On emergency admission to Hospital Médica Sur, a tertiary care private institution in Mexico City, on December 8, 2025, the patient was awake but disoriented, with marked fluctuations in attention. Her speech was dysarthric and incoherent. She had autonomic instability, with tachycardia (131 bpm) and hypertension (149/91 mmHg). Cranial nerve examination revealed orofacial dyskinesias without clear focal deficits. Motor examination demonstrated hyperreflexia, increased muscle tone, and focal weakness of the right upper limb. Sensory examination was limited because of poor cooperation; however, no clear asymmetries were identified.

**Figure 1:**
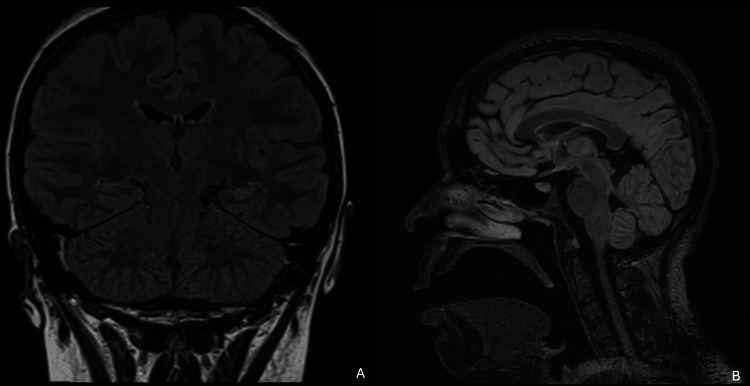
Brain MRI. Brain MRI demonstrating no structural abnormalities. (A) Coronal fluid-attenuated inversion recovery (FLAIR) sequence. (B) Sagittal T2-weighted sequence.

Brain MRI demonstrated no structural abnormalities, including abscesses, neoplasms, or vascular lesions. In the absence of focal lesions, mass effect, or limbic signal abnormalities suggestive of a primary structural central nervous system process, an inflammatory or nonstructural etiology was suspected (Figure 1).

**Figure 2:**
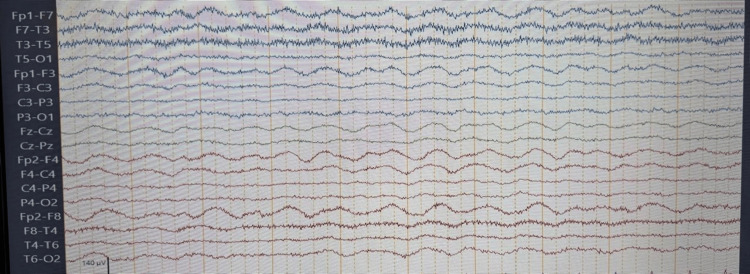
EEG demonstrating diffuse slowing with delta-brush-like activity. Longitudinal bipolar electroencephalography (EEG) demonstrating diffuse high-amplitude delta slowing with superimposed low-amplitude fast activity, forming a delta-brush–like pattern with frontocentral predominance. No definite epileptiform discharges are identified in this segment. These findings suggest diffuse cortical dysfunction and may be seen in patients with anti-NMDA receptor encephalitis in the appropriate clinical context.

CSF analysis revealed mild lymphocytic pleocytosis with normal glucose and protein levels. Infectious studies, including CSF, blood, and urine cultures, were negative. Electroencephalography (EEG) demonstrated diffuse theta-delta slowing with superimposed irritative activity, consistent with moderate-to-severe cortical-subcortical dysfunction. Extreme delta brush was not observed (Figure 2).

Although nonspecific, these findings supported the presence of an encephalopathic process within the broader clinical context. The generalized slowing pattern was interpreted as supportive evidence of diffuse cerebral dysfunction, which may be observed in autoimmune encephalitis.

At this stage, the differential diagnosis included infectious encephalitis, other forms of autoimmune encephalitis, and primary psychiatric disorders. Infectious etiologies were considered less likely given the absence of fever, a negative comprehensive PCR panel, and only mild inflammatory changes in the CSF. Conversely, primary psychiatric disorders were considered unlikely because of the presence of seizures, autonomic instability, and objective neurological deficits. The overall clinical presentation, together with CSF inflammatory findings and the identification of an ovarian teratoma, was considered highly suggestive of autoimmune encephalitis.

The patient’s mother reported a 10-month history of a 2 cm ovarian cyst that had been managed with oral contraceptives under gynecologic supervision. A comprehensive gynecologic oncology evaluation was subsequently performed, including pelvic MRI, which demonstrated a left ovarian mass measuring 11 × 14 × 5.4 cm (estimated volume: 432 mL), with heterogeneous cystic components, extensive fatty elements, and a mural nodule. The lesion was classified as O-RADS 2 and was highly suggestive of a mature cystic teratoma.

Tumor markers, including alpha-fetoprotein, CA-125, CA 19-9, and carcinoembryonic antigen, were within normal limits. Given the strong clinical suspicion of paraneoplastic encephalitis after exclusion of other infectious and structural etiologies, the patient was screened for anti-NMDA receptor antibodies, with results confirming the diagnosis on day 14. Laboratory findings are summarized in Tables 1-2.

**Table 1:** Initial laboratory findings. AFP: Alpha-fetoprotein; CA-125: Cancer antigen 125; CA 19-9: Carbohydrate antigen 19-9; CEA: Carcinoembryonic antigen; ANA: Antinuclear antibodies; anti-dsDNA: Anti-double-stranded DNA antibodies; PT: Prothrombin time; INR: International normalized ratio. Reference ranges correspond to standard adult values.

Parameter	Value	Units	Reference range
Anti-NMDA receptor antibodies	Positive (↑)	-	Negative
Alpha-fetoprotein (AFP)	2.1	ng/mL	<10
CA-125	6	U/mL	<35
CA 19-9	4.9	U/mL	<37
Carcinoembryonic antigen (CEA)	0.97	ng/mL	<5
Leukocytes	13.32 (↑)	×10³/µL	4.0-10.0
Sodium	140	mEq/L	135-145
Fibrinogen	<50 (↓)	mg/dL	200-400
ANA	Negative	-	Negative
Anti-dsDNA	10.6	IU/mL	<30
Lupus anticoagulant	Negative	-	Negative

**Table 2:** Initial cerebrospinal fluid findings.

Parameter	Value	Units	Reference range
CSF leukocytes	11 (↑)	cells/mm³	0-5
CSF glucose	63	mg/dL	45-80
CSF protein	39.9	mg/dL	15-45
CSF infectious PCR	Negative	-	Negative

High-dose IV methylprednisolone (1 g/day for five consecutive days) was initiated early as first-line immunotherapy. Due to persistent severe symptoms, therapeutic plasma exchange (PLEX) was administered every 48 hours for a total of five sessions, resulting in transient hypofibrinogenemia and coagulopathy without bleeding complications. PLEX was selected to facilitate the rapid removal of circulating pathogenic antibodies.

**Figure 3:**
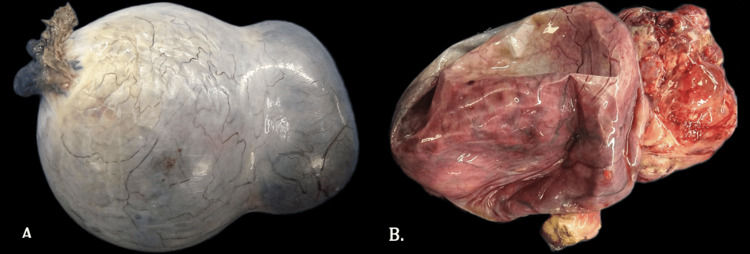
Macroscopic appearance of a mature ovarian teratoma. (A) External surface: The specimen weighs 614 g and measures 10.2 × 8.4 cm. (B) Internal surface: A multiloculated cystic lesion with a mural nodule on the right side, cartilaginous-appearing areas, sebaceous components, and cutaneous adnexal structures.

**Figure 4:**
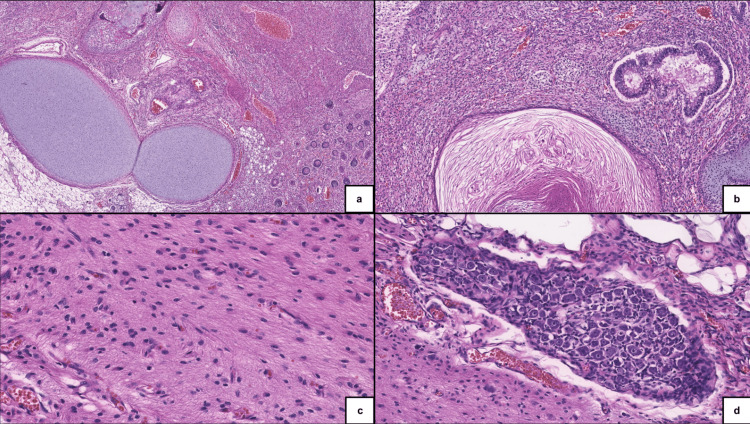
Microscopic features of a mature ovarian teratoma. (A) Mesenchymal components, including hyaline cartilage, osseous formation, and mature adipose tissue associated with hair follicles. (B) Glandular structures and keratinization. (C) Neuroglial component. (D) Ganglion cells.

**Figure 5:**
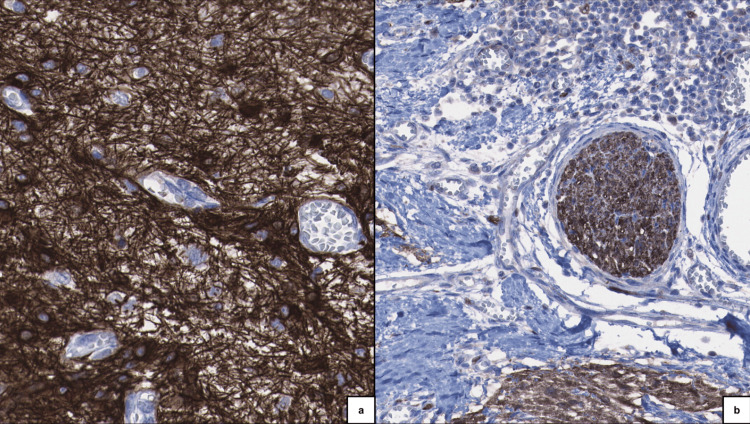
Immunohistochemical staining. (A) Glial fibrillary acidic protein (GFAP) demonstrating immunoreactivity in glial cells/astrocytes. (B) S100 protein expression in the neural component, highlighting neural processes and a nerve in transverse section.

In view of the strong correlation between ovarian teratomas and the development of anti-NMDA receptor encephalitis, early surgical resection was prioritized owing to continuous autonomic instability and neurological decline on the third day of ICU admission. The patient underwent exploratory laparotomy with left salpingo-oophorectomy and peritoneal cytology. Histopathology confirmed a 10.2 × 8.4 cm mature cystic teratoma without malignant transformation (Figures 3-5).

Due to persistent severe neurological involvement despite first-line therapy and tumor resection, intravenous immunoglobulin (IVIG) (2 g/kg administered over five days) was subsequently initiated to provide sustained immunomodulation. In addition, second-line immunotherapy with rituximab (375 mg/m² weekly for two doses) was started, targeting CD20-positive B lymphocytes responsible for ongoing antibody production.

At the time of discharge, after five weeks of inpatient management, the patient was alert, oriented, and ambulatory with minimal assistance while continuing outpatient neurological rehabilitation. Continued clinical improvement was documented over the following three months of follow-up, with only minimal residual weakness in the upper extremities.

## Discussion

Anti-NMDA receptor encephalitis represents a paradigmatic model of a surgically modifiable autoimmune neurologic disorder when associated with a synchronous tumor, a phenomenon occurring in approximately 40 to 59% of cases [2,8]. Antibody-mediated reversible synaptic dysfunction explains the substantial recovery observed with timely immunotherapy and tumor removal. The most common associated neoplasms include ovarian teratomas (94%), followed by extraovarian teratomas (2%), and, rarely, other epithelial tumors such as lung, breast, testicular, pancreatic, and thymic carcinomas [5].

The present case illustrates the complex diagnostic and therapeutic course of this entity, emphasizing the pivotal role of tumor identification and surgical resection within a multidisciplinary management strategy, even before antibody confirmation.

The initial presentation of this patient was dominated by rapidly progressive neuropsychiatric symptoms, seizures, movement disorders, and autonomic instability, a pattern consistent with previously described clinical trajectories of anti-NMDA receptor encephalitis [1-3]. As frequently reported, early neuroimaging is often normal or nonspecific, and CSF analysis may show only mild inflammatory changes, underscoring the diagnostic challenge and the importance of antibody testing in both serum and cerebrospinal fluid [4]. Electroencephalographic abnormalities reflecting diffuse cortical-subcortical dysfunction further supported the diagnosis. Management was guided by a rigorous diagnostic synthesis. Initial considerations included infectious, autoimmune, and primary psychiatric etiologies. Infectious causes were considered unlikely because of negative microbiological testing and unremarkable CSF findings. Although multiple autoimmune encephalitides were included in the differential diagnosis, the specific combination of psychiatric features, seizures, and dyskinesias favored anti-NMDA receptor encephalitis. The discovery of an ovarian teratoma further increased the pretest probability of a paraneoplastic process, facilitating the decision for prompt surgical resection before antibody confirmation.

Ovarian teratomas act as the antigenic driver of the autoimmune response because of the presence of ectopic neural tissue expressing NMDA receptors. Surgical resection should be considered a time-sensitive neurological intervention rather than solely an oncologic procedure. Early tumor removal has been independently associated with faster neurological recovery and lower relapse rates [9]. In the large multicenter cohort reported by Titulaer MJ et al., which included 577 patients, early resection of the associated tumor (less than 1.4 months) was identified as an independent predictor of favorable outcome. Patients undergoing prompt surgery showed earlier neurological improvement, with substantial recovery typically occurring within the first three to six months and continued functional gains over an 18-month follow-up period [5]. Similarly, in the expanded series by Dalmau J et al., patients who underwent early tumor removal within four months demonstrated earlier clinical stabilization, with progressive neurological recovery beginning at a median time of eight weeks (range: 2-24 weeks) after surgery, whereas delayed or absent tumor resection was associated with a prolonged disease course, with a median time of 11 weeks (range: 2-50 weeks) [2,8].

In a Chinese referral center, a prospective cohort of 31 female patients diagnosed with anti-NMDA receptor encephalitis associated with suspected ovarian teratoma was analyzed from 2011 to 2016. All patients underwent laparoscopic evaluation, with histologically confirmed ovarian teratoma in 29 patients (93.5%), one of whom was diagnosed with a grade 1 immature ovarian teratoma. One fatality (3.4%) occurred due to severe infection and respiratory failure secondary to prolonged seizures. Surgical resection of the ovarian teratoma was associated with a reduced risk of relapse. Notably, none of the 29 patients with confirmed teratomas presented with pre-onset lower abdominal pain or acute ovarian torsion. Consequently, these findings suggest that pelvic ultrasonography is essential for all female patients with anti-NMDA receptor encephalitis to effectively screen for underlying ovarian teratomas [10].

In a systematic review of 174 cases of anti-NMDAR encephalitis associated with ovarian teratoma, Acién P et al. found that a prolonged interval between diagnosis and tumor removal correlated with longer intensive care unit stays and delayed neurological recovery. The median time to surgery was 28 days (range: 2-455 days); however, for mature teratomas, it was 71.4 ± 88.5 days, largely because of low clinical suspicion among neurologists and psychiatrists, with only 6% of cases receiving early evaluation by gynecology or oncology services [6]. Few case reports exist in Mexico; Jiménez-Ruiz A et al. described a 20-year-old Mexican woman with a presentation similar to that of the present case. However, they did not report the timing of the unilateral right oophorectomy, with neurological improvement observed at discharge after 35 days and return to daily activities at the one-year follow-up [11].

In this case, tumor resection was performed within 72 hours of hospital admission, balancing the risks of operative intervention against the need to eliminate the antigenic source. This intervention was prioritized because the patient exhibited persistent severe autonomic instability secondary to dysautonomia despite initial immunotherapy. Through multidisciplinary collaboration between the intensivist and oncology anesthesiology teams, favorable operative outcomes were achieved; the requirement for vasoactive support decreased within the initial postoperative hours, and mechanical ventilation was not required.

Gradual neurological improvement was observed during the following two weeks after multidisciplinary management in the ICU, with significant functional recovery at discharge after five weeks. The favorable neurological trajectory observed thereafter supports the concept that tumor resection appears to play a disease-modifying role, as described in other observational cohorts [6-8]. Although anti-NMDA receptor antibody confirmation was pending at the time of surgery, clinical suspicion remained high because differential diagnoses, including infectious, autoimmune, and primary CNS neoplastic processes, had been ruled out. The delay in antibody confirmation was due to the logistical requirement of transporting samples to the Mayo Clinic, which carries a 10- to 14-day processing time.

The therapeutic course highlights the stepwise escalation recommended in current guidelines. First-line immunotherapy, including high-dose corticosteroids, therapeutic plasma exchange, and intravenous immunoglobulin, was initiated early in the disease course. Despite partial stabilization, the persistence of severe neurological dysfunction required further escalation to second-line treatment with rituximab, a strategy supported by evidence demonstrating improved outcomes in refractory or severe cases, as reported by Titulaer MJ et al. [5,12]. Favorable prognostic factors in this case included young age, early immunotherapy, timely tumor resection, and escalation to second-line therapy, with a lower likelihood of relapse. Nosadini M et al. demonstrated in a meta-analysis that delayed immunotherapy administration beyond 30 days after neurological symptom onset, or failure to administer immunotherapy, was associated with a 2.7-fold increased odds ratio for worse outcomes, highlighting the importance of immunotherapy in the management of this disease [13].

Relapse occurs in approximately 12-24% of patients, particularly in those without tumor removal. Therefore, long-term neurological follow-up is essential to monitor for recurrence and cognitive sequelae [14,15].

This case serves as a novel contribution to the literature on Latin American patients with anti-NMDA receptor encephalitis. It emphasizes the critical role of early surgery in determining neurological prognosis and supports the feasibility and apparent safety of intervention within a 72-hour window in carefully selected patients. This report represents an uncommon documentation of such rapid surgical management and its subsequent favorable clinical outcomes.

From an oncologic-surgical standpoint, awareness of this entity is essential, as timely tumor identification and resection can substantially alter the natural history and neurological prognosis of the disease.

## Conclusions

Anti-NMDA receptor encephalitis is a potentially reversible yet life-threatening condition. In young women, systematic oncologic evaluation is essential, and early surgical resection of associated ovarian teratomas has been associated with improved neurological outcomes when combined with timely, stepwise immunomodulatory therapy.

Although limited by its nature as a single case report, this contribution addresses a significant gap in Latin American clinical data. The successful outcome reinforces the importance of a multidisciplinary approach to early detection and treatment. We conclude that such integrated collaboration is fundamental to optimizing therapeutic interventions, supporting the safety of very early surgical resection in carefully selected patients, and improving neurological prognosis.
